# Knowledge mapping of targeted immunotherapy for myasthenia gravis from 1998 to 2022: A bibliometric analysis

**DOI:** 10.3389/fimmu.2022.998217

**Published:** 2022-09-29

**Authors:** Yue Su, Zhe Ruan, Rui Wang, Sijia Hao, Yonglan Tang, Xiaoxi Huang, Ting Gao, Zhuyi Li, Ting Chang

**Affiliations:** ^1^ Department of Neurology, Tangdu Hospital, The Fourth Military Medical University, Xi’an, China; ^2^ Medical Department of Tangdu Hospital, The Fourth Military Medical University, Xi’an, China

**Keywords:** myasthenia gravis, bibliometric, VOSviewer, Citespace, targeted immunotherapy

## Abstract

**Background:**

The treatment of myasthenia gravis (MG) has advanced from steroids and traditional immunosuppressants to targeted immunotherapy. Targeted immunotherapy has been successfully employed in clinical practice in recent years. This study aimed to explore the emerging trend of targeted immunotherapy in MG and summarize the knowledge structure through bibliometric methods.

**Methods:**

The Web of Science Core Collection database (WoSCC) was chosen to retrieve the literature on targeted immunotherapy for MG. Two bibliometric analysis software, VOSviewer and CiteSpace, and bibliometric online platform were mainly used to evaluate the contributions from countries/regions, institutions, journals, and authors through the construction and visualization of bibliometric networks. By systematically reviewing a knowledge domain, future research developments were determined. The R version 4.1.2 and Microsoft Excel 365 were used for statistical analysis.

**Results:**

A total of 562 original articles and 262 reviews relevant to MG targeted immunotherapy were included. The number of publications on targeted immunotherapy for MG exhibited a two-phase advancement. The first stage showed a steady growth trend from 1998 to 2016, with an annual number of no more than 35 publications. The second stage revealed an explosive growth trend from 2017, reaching a peak number of publications in 2020. The United States ranked first in the number of publications, citations, and h-index. The author with the highest citation and h-index was Vincent A. And 28.03% of the articles were published in the top 10 journals. In addition to “myasthenia gravis”, the keyword with the highest consideration was “rituximab”, followed by “double-blind”, which indicate research hotspots gradually from basic research to clinical research over time, especially in the field of targeted immunotherapy. The MG treatment has entered a personalized precision treatment phase. Exploration into new target molecules and conducting high-quality randomized controlled trials on existing biological agents are the further research direction.

**Conclusion:**

The current study summarized the global research trends concerning targeted immunotherapy for MG. Research interests gradually advanced from basic research to clinical research. MG treatment has entered a personalized precision treatment phase. Further investigations into new target molecules and high-quality randomized controlled trials on existing biological agents are required urgently to direct future immunotherapy research.

## Introduction

Myasthenia gravis (MG) is an acquired autoimmune disease, manifested by disruption of neuromuscular junction (NMJ) transmission caused by autoantibody, cellular immune dependence, and complement (1). Anti-acetylcholine receptor antibodies (AChR-Ab) are the frequent cause of pathogenesis, and the main clinical manifestations are fluctuating skeletal muscle weakness and fatigue (2). The global annual incidence of MG is 0.4-1 per 100,000 people and the worldwide prevalence was 15-25 per 100,000 people (3–5). Presently, the treatment of MG is based on steroidal and other traditional immunosuppressants, such as tacrolimus, azathioprine, and mycophenolate mofetil (6). Nevertheless, long-term use of steroids can cause serious side effects such as abdominal obesity, elevated blood pressure, elevated plasma glucose level, osteoporosis, and necrosis of the femoral head (7). The traditional immunosuppressive agents not only have a slower onset of effect but are associated with the risk of tumorigenesis, myelosuppression, and infection. Therefore, these side effects will further burden the disease. Furthermore, the selection of MG treatment remains very challenging due to the heterogeneity in pathogenesis, clinical manifestations, and drug reactions (1). Targeted biological agents are a class of small molecule inhibitors that specifically target inflammatory cytokines, immune cells, and intracellular kinases (8). The clinical use of these drugs has changed the treatment landscape for autoimmune diseases. A variety of targeted biological agents targeting immune cells, complements, neonatal Fc receptors, and cytokines have entered phase II and III clinical trials (9–12). These targeted biological agents can alleviate the clinical symptoms quickly, significantly, and continuously with favorable tolerability and safety (13). These targeted biological agents can reduce the dosage of steroids and accelerate precision medicine. Therefore, the targeted biological agents possess significant research value and promising clinical applications.

Bibliometric analysis and data visualization, a well-established bioinformatics tool, are used to analyze a field of research quantitatively and qualitatively, provide evidence for the impact of an area of research, find the emerging area of research, and identify potential research collaborators (14, 15). Different from meta-analysis and systematic review, bibliometric analysis integrates information visualization techniques with mathematical and statistical analyses to assess institutions performing research, contributing authors, journals publishing a specific area of research, and countries/regions with a research area of interest (16). It primarily evaluates the characteristics of the literature, such as the number of publications, citations, and research or clinical collaborations (17, 18). These analyses provide guidelines for assessing research trends and developing research areas (19). This analytical method of the literature is used in all areas of basic and clinical research.

A team published high-impact articles on targeted immunotherapy for MG. Who are the core authors of these studies? Who are their collaborators? What research topics were they interested in? Which topic received the most attention? What journals were they published in? How did the specific research area develop and evolve? No individual can read all the high-impact articles on a specific area due to limited time and energy (20). Therefore, bibliometrics provides a new method for literature analysis, so that readers can rapidly understand the emerging subjects in their research areas of interest and read the selected literature (21, 22). Researchers can use these data to quickly identify potential new collaborators in their respective research fields.

No bibliometric studies have been published on targeted immunotherapy for MG thus far. This paper aimed to systematically summarize and visually analyze the literature in the field of targeted immunotherapy for MG based on the Web of Science and using CiteSpace and VOSviewer software to understand the frontiers and emerging trends of research. The outcome can provide more references, novel insights, and directions for future clinical research and guidelines establishments. This bibliometric analysis is the first attempt relevant to this area of research.

## Methods

### Data source

There were only a few articles on MG targeted immunotherapy before 1998, while after rituximab was used for treatment MG in 2000 firstly (23), the articles on MG targeted immunotherapy gradually increase over time. Therefore, the Science Citation Index Expanded (SCI-Expanded, 1998-present) of the Web of Science Core Collection (WoSCC) database was selected after considering the limitations and strengths of diverse databases (24). The Web of Science (WoS) was created by Thomson Scientific to make citation indices (that E. Garfield assessed since the early 1960s) accessible *via* the internet, which is the oldest citation database and is currently owned by Clarivate Analytics Company (Philadelphia, United States of America) (24). The selection to use the WoSCC database was justified for the following reasons. First, the WoSCC is the most comprehensive and authoritative database when compared with other databases, such as PubMed, Scopus, and Embase (25, 26). Second, it is a classic citation database, including literature abstracts and other relevant data, such as citations and research collaboration information, which is useful for bibliometric analysis (17). Finally, it can directly provide reference information that is required for the construction and visualization of bibliometric networks by VOSviewer and CiteSpace. Otherwise, an additional operation is required to change the file format if the information is retrieved from another database. Therefore, the WoSCC is considered the most suitable online database for bibliometric analysis (27–29).

### Retrieval strategies

**Figure 1:**
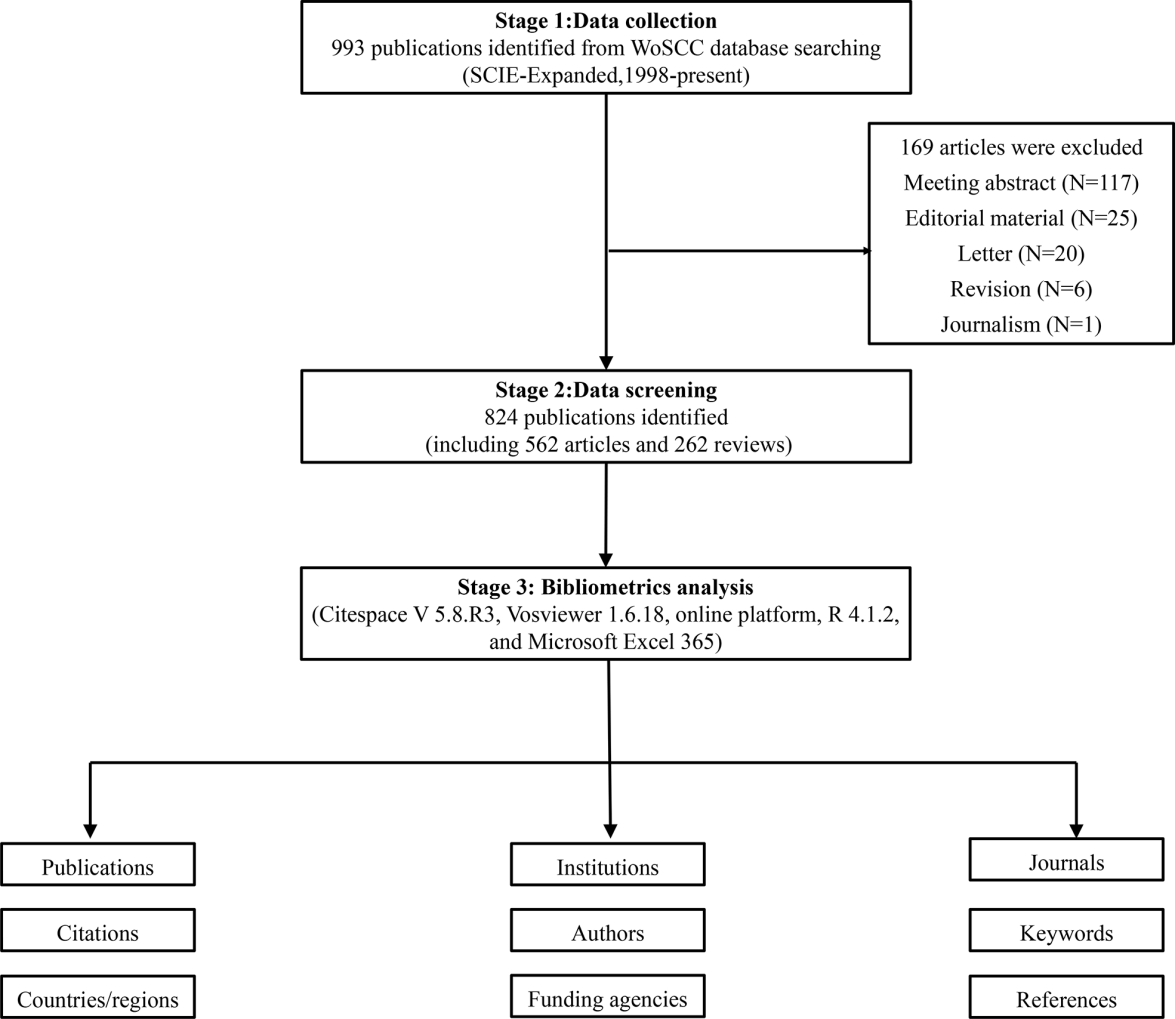
Flowchart of the literature search and selection process.

The advanced retrieval function was used to improve the quality of information. The specific retrieval rules were as follows: #1: TS= (myasthenia gravis); #2: TS=(eculizumab) OR TS=(rituximab) OR TS=(RTX) OR TS=(tocilizumab) OR TS=(belimumab) OR TS=(rozanolixizumab) OR TS=(efgartigimod) OR TS=(Zilucoplan) OR TS= (monoclonal antibody) OR TS= (biologic drugs) OR TS= (targeted immunotherapy) OR TS= (targeted immunotherap*) OR TS= (novel therap*) OR TS= (novel treatment strategies); the ultimate dataset: #1 AND #2. Literature in English only were included. The search was limited to systematic reviews and original articles. A truncation symbol “*” was used and the use of truncation searches improved recall and prevented missing inspection. All contents including the titles, authors, abstracts, keywords, and cited references were recorded. A total of 993 records on targeted immunotherapy for MG were searched from 1998 to 2022 (retrieved on April 25, 2022). The exclusion materials were 169 records including meeting abstract, editorial material, revision, letters, journalism, and non-English works of literature. Consequentially, 824 valid literatures (562 articles and 262 reviews) were retrieved as the final dataset and exported in the form of “full record and cited references” for further analysis. Subsequently, the text files were renamed as “download∗.txt”, which were recognized by CiteSpace software. The detailed literature screening process is shown in **Figure 1**.

### Data extraction

These data were imported into Microsoft Excel 365 (Microsoft Corporation, Redmond, Washington, WA, United States) for further processing. Two researchers (YS and ZR) performed data extraction and literature selection and analysis to ensure the reliability of the results independently. Any discrepancies between the two researchers were discussed to reach a consensus. The disagreements were resolved through discussion or a third-party consultation (RW and SH). The indicators such as the annual number of publications and citations, countries/regions, journals, institutions, authors, co-cited references, and keywords were primarily focused on. The citation reporting of the WoSCC was used to assess the h-index and citation frequency. The h-index was calculated considering a scientist/country has published h articles, each of which has been cited at least h times (30). This index was typically used to assess the scientific impact and productivity of a researcher/country (31). The journal impact factor (IF) and category (Q1, Q2, Q3, or Q4) were retrieved from the Journal Citation Reports (JCR) 2021, which is the most widely used reference standard for evaluating the journal performance within its field.

### Data visualization and analysis

Three bibliometric tools, including two software (CiteSpace (5. 8. R3) and VOSviewer (1. 6. 18)) and an online platform were used in this study for a more comprehensive analysis.

### VOSviewer

VOSviewer (version 1.6.18, the Netherlands, downloaded from http://vosviewer.com) is a literature knowledge visualization software that uses the Visualization of Similarities (VOS) technology, which was developed by Professors Eck and Waltman from Leiden University using the Java language. The VOSviewer assesses and visualizes research characteristics from different perspectives, such as co-authors, research institutions, countries/regions, keywords, and co-cited references (32). In the network visualization map, each node corresponds to parameters, such as countries/regions, institutions, journals, authors, or keywords. The diameter of its size is roughly proportional to the number of publications, citations, or occurrences. Closer terms in the same publications are automatically assigned to a cluster with the same color. Otherwise, the nodes are set apart with different color coding. The link between nodes represents the network connection and the strength of the link. The total link index is used to quantitatively evaluate the strength (TLS), which is the sum of the link strength of all other terms (26, 27). Furthermore, the VOSviewer can provide three types of network maps, including the network visualization map, the overlay visualization map, and the density visualization map (33).

### CiteSpace

CiteSpace (Version 5.8. R3, downloaded from https://sourceforge.net/projects/citespace/) is a Java-based computer program designed by professor Chen from Drexel University (34), and it is an influential visualization software to obtain quantitative information and discover the related development trends and dynamics in a particular scientific research field (34). The network maps generated by the CiteSpace were also composed of links and nodes. The nodes normally represent the authors, country/regions, or institutions, whereas links represent co-authorship between these nodes. The centrality is an important indicator that unveils the importance of a node in the network, and the higher the centrality the node has, the larger the impact the node has on the map (35). The burst detection of references and keywords recognizes the sharp increases in scientific activities over a limited period and captures the increasing interest in a specific research field (36).

### Bibliometric analysis using an online platform

In addition to the above methods, an online platform for bibliometric analysis and visualization, https://bibliometric.com/ (accessed on 28 April 2022), plotted the distribution and international collaboration of countries/regions.

### Materials and methods ethics statement

This study did not involve human or animal subjects and all data used in this manuscript were obtained from public databases. Therefore, ethical approval was not required.

### Statistical analysis

Statistical analysis was performed using R version 4.1.2 (The R Foundation for Statistical Computing, Vienna, Austria) and Microsoft Excel 365 (Microsoft Corporation). The VOSviewer (version 1.6.18, Leiden University, Leiden, the Netherlands) and CiteSpace (version 5.8.R3, Drexel University, Philadelphia, PA, USA) were used for the analysis of basic metrics.

## Result

### The growth trend of publication outputs and citations

**Figure 2:**
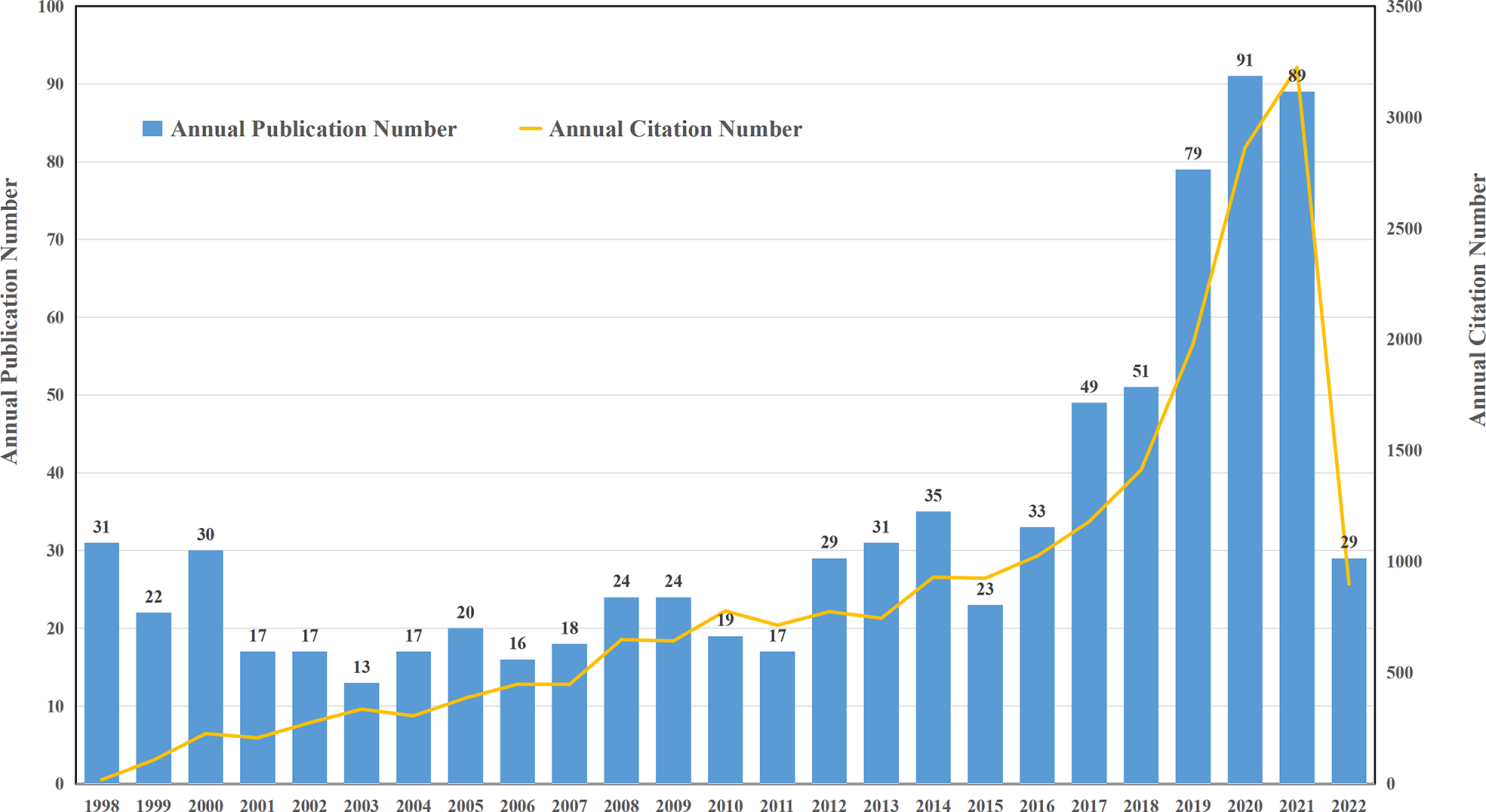
Global trend of annual publications and citations related to targeted immunotherapy for MG from 1998 to 2022.

Quantitative analysis of the published papers can reflect the productivity of a given scientific research field over the years and exhibit the trend in development in a specific area. Utilizing the aforementioned search strategies, a total of 993 articles were retrieved. After excluding invalid articles, 824 publications, including 562 original research articles and 262 reviews were included in the final analysis (**Figure 1**). The annual distribution of publications and total citations of annual publications from 1998 to 2022 are shown in **Figure 2**. During the past 24 years, with the exception of decrease in number at some time points, the annual number of articles on targeted immunotherapy for MG has shown a steady growth trend and reached its peak in 2020.There were two growth phases according to the curve: an early stationary growth phase from 1998 to 2016 and a rapid-growing phase from 2017 to 2022. Based on the WoSCC database analysis, 34.95% of them (824) were published in the last four years, all publications related to targeted immunotherapy for MG have been cited 22184 times (18346 times after the removal of self-citations) with 26.92 citations per paper and the H-index of 70.

### Analysis of published articles by countries/regions

**Figure 3:**
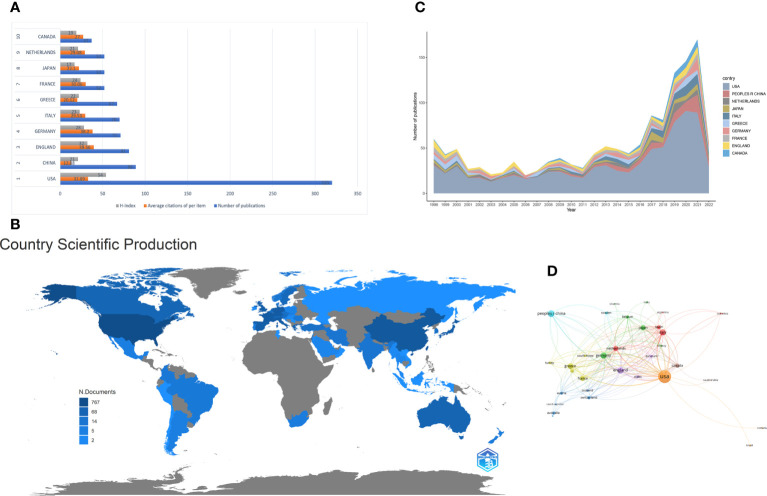
**(A)** The number of publications, average citations of per items and H-index of the top 10 countries/regions. **(B)** Geographic distribution map displaying the global distribution of targeted immunotherapy for MG. Different countries/regions were denoted with different colors based on the number of articles published. **(C)** The annual number of publications from the top 10 countries/regions between 1998 and 2022. **(D)** Citation map of countries/regions on targeted immunotherapy for MG generated by the VOSviewer. Each node represents a country/region, and node size indicates the number of publications. The connection between the nodes represents a citation relationship, and the thickness of the lines indicates citation strength (weights on the TLS).

**Figure 4:**
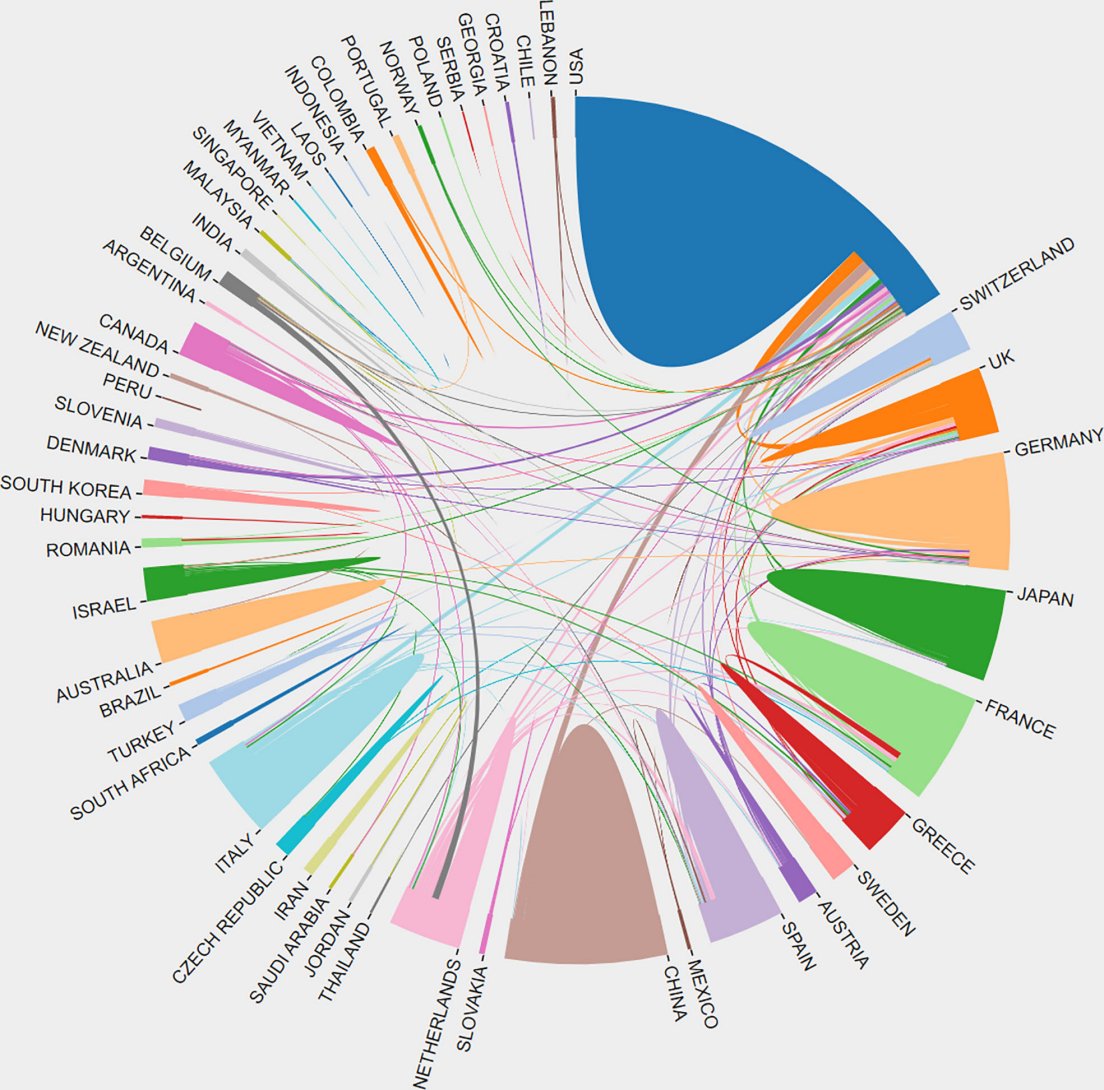
Distribution and international cooperation of countries/regions that are involved in targeted immunotherapy for MG. The thickness of the line reflects the frequency of the cooperation. The thicker the line, the stronger the cooperation.

**Table 1** summarizes these publications from the top 10 countries/regions. The retrieved articles were from 59 countries/regions. The USA ranked first in research productivity [320 (38.83%)], followed by China [89 (10.80%)], England [81 (9.83%)], Germany [71 (8.62%)], and Italy [70 (8.50%)]. After removing self-citations, the USA had 9696 citations and an h-index of 54. Both parameters ranked first among all countries/regions analyzed, followed by England, Germany, Italy, and France. The overall citations and h-index were 3097, 2665, 1979, and 1524 and 32, 28, 23, and 24, respectively (**Figure 3A**). The geographical distribution map based on the total number of publications from the distinct country is shown in **Figure 3B**. On the map, the lighter colors represent the low density of publications, and the darker colors represent the high density. Annual trends in the number of articles are displayed in **Figure 3C**, and the USA was the leading country in the annual number of publications from 1998 to 2022. A collaboration analysis was conducted to examine the international collaboration observed from 1998 to 2022. **Figure 4** demonstrates that the USA had the greatest international collaboration in this area followed by China. The United Kingdom has the strongest connection with the USA. Links represent international collaboration pathways between countries. Only countries/regions with a minimum number of 3 publications were included in the network. Only 34 countries/regions that met the threshold were analyzed using the VOSviewer (**Figure 3D**). There were 34 nodes, 8 clusters, and 176 links on the network map. The top three countries with the largest TLS were the USA (TLS =200), England (TLS = 129), and Germany (TLS = 99).

**Table 1:** The top 10 countries/regions contributing to targeted immunotherapy for MG.

Rank	Country/Region	Number of publications	Number of citations	Citations per article	H-Index
1	USA	320	9696	32.89	54
2	CHINA	89	1146	12.88	21
3	ENGLAND	81	3097	39.56	32
4	GERMANY	71	2665	38.2	28
5	ITALY	70	1979	29.53	23
6	GREECE	67	1193	20.12	22
7	FRANCE	52	1524	30.06	24
8	JAPAN	52	1112	22.1	17
9	NETHERLANDS	52	1451	29.08	21
10	CANADA	37	937	27	19

### Analysis of the institutions with the most productivity

**Figure 5:**
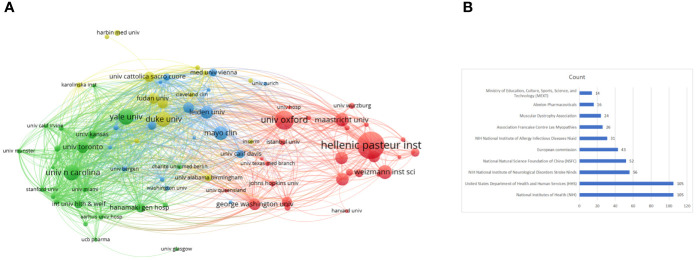
**(A)** Network visualization of the institution co-citation analysis on targeted immunotherapy for MG based on the VOSviewer. **(B)** The top 10 funding agencies for the output of targeted immunotherapy for MG.

A total of 1084 institutions published scientific articles on targeted immunotherapy for MG during the defined study period. As shown in **Table 2**, the top 10 institutions accounted for 323 (39.20%) of literatures in this field, and the League of European Research Universities was the largest contributor in terms of numbers of publications with 81 (9.83%) articles, followed by the University of Oxford with 40 (4.85%) articles and the Hellenic Pasteur Institution with 35 articles (4.25%). The institution citation analysis is presented in **Figure 5A**. The publications originating from 81 institutions were selected, with a minimum number of documents of more than 5 from each country. The data were analyzed by using the VOSviewer and there were 81 nodes, 4 clusters, and 1862 links on the network map, the hellenic pasteur institution at the center of node.

**Table 2:** The top 10 institutions with most publications in the field of targeted immunotherapy for MG.

Rank	Institution	Number of publications	Number of citations	Citations of per article	H-Index
1	League of European Research Universities Leru	81	3467	44.05	32
2	University of Oxford	40	1631	41.9	20
3	Hellenic Pasteurinst	35	595	19.46	15
4	Udice French Research Universities	26	853	33.15	16
5	University of California System	26	677	26.5	12
6	Yale University	26	849	34.46	14
7	Maastricht University	20	495	25.95	11
8	University of Texas System	25	518	21.56	13
9	University of North Carolina	23	795	36.48	13
10	University of North Carolina Chapel Hill	21	763	38.38	13

### Analysis of funding agencies

As noted above, the economic foundation contributed the most to scientific development. A summary of the top 10 most active funding agencies in this area is provided in **Table 3** and **Figure 5B**. The funding organizations from the USA including the National Institutes of Health (NIH), United States Department of Health and Human Services (HHS), and NIH National Institute of Neurological Disorders Stroke (NIDS) occupied the top three positions in contributing to this field, and funded 105, 105, and 56 studies, respectively. The remaining funding agencies were from China, Belgium, France, and Japan. In addition to having well-established institutions, the USA maintained its leading position in the domain of targeted immunotherapy for MG, which was not separated from the support of adequate funding. Adequate funding can attract a wider variety of researchers and institutions to devote more work to this area, which is a mutually reinforcing process.

**Table 3:** The top 10 funding agencies contributed to targeted immunotherapy for MG.

Rank	Funding agencies	Countries	Count	Percentage(N=824)	H-index
1	National Institutes of Health (NIH)	USA	105	12.74	37
2	United States Department of Health and Human Services (HHS)	USA	105	12.74	37
3	NIH National Institute of Neurological Disorders Stroke (NINDS)	USA	56	6.80	25
4	National Natural Science Foundation of China (NSFC)	China	52	6.31	12
5	European commission	Belgium	43	5.22	21
6	NIH National Institute of Allergy Infectious Diseases (NIAID)	USA	31	3.76	20
7	Association Francaise Contre Les Myopathies	France	26	3.16	15
8	Muscular Dystrophy Association	USA	24	2.91	17
9	Alexion Pharmaceuticals	USA	16	1.94	9
10	Ministry of Education, Culture, Sports, Science, and Technology (MEXT)	Japan	14	1.70	8

### Analysis of journals and co-cited journals

**Figure 6:**
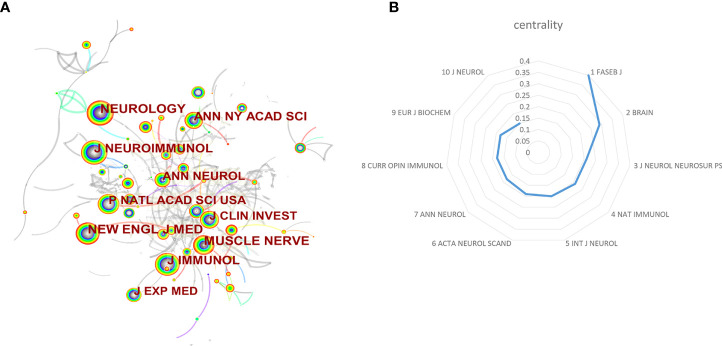
**(A)** Visualization map of journal co-citation analysis by using CiteSpace. **(B)** The top 10 centrality of journals for the targeted immunotherapy for MG.

**Figure 7:**
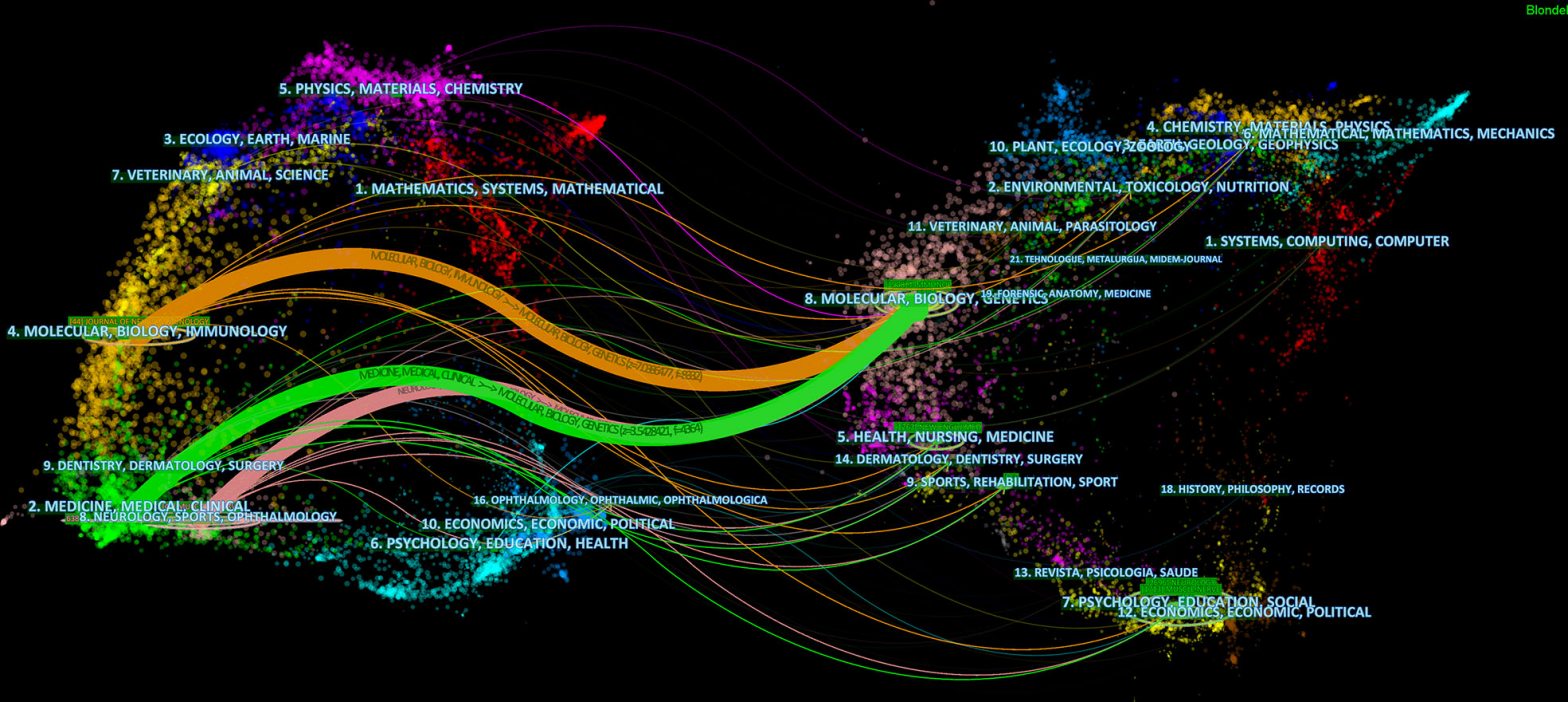
The dual-map overlay of academic journals in the field of targeted immunotherapy for MG based on the CiteSpace software. The labels represent different research subjects covered by the journals. The citing journals are on the left side, while the other side of the map represents the cited journals. Different colored lines correspond to the different paths of references, beginning with the citing map and ending at the cited map. The path widths are scaled proportionally to the frequency of z-score-scale citation.

In total, the retrieval article was published in 323 journals in this research field. The top 10 active journals that published 231 papers on targeted immunotherapy for MG, accounted for 28.03% of all 824 publications. **Table 4** summarizes the basic information on the top 10 journals. The highest number of relevant articles were published in the *Journal of Neuroimmunology* [44 (5.34%)], and *Muscle Nerve* [41 (4.98%)] ranked second, followed by *Annals of the New York Academy of Sciences* [27 (3.28%)]. According to the 2020 JCR standards, the IF of the top 10 journals ranged from 3.217 (*Muscle Nerve*) to 9.91 (*Neurology*) and was classified as Q1 to Q2 categories. In addition to the number of publications, the impact factor of journals also depends on how often they are co-cited in a particular field of research. As shown in **Figures 6A, B**, co-citation analysis was performed by the CiteSpace software to determine the connection between journals that were cited in other journals, and there were 300 nodes and 448 links in the co-cited network map. The *FASEB J* had the highest centrality, with a central value of 0.4, followed by the *Brain* (0.29) and *Nat Immunol* and *J Neurol Neurosur Ps* (0.2). Additionally, a dual map overlay of the journals on targeted immunotherapy for MG was constructed (**Figure 7** **)**. The dual map overlay of journals described the topic distribution of academic journals, and the map of the citing journals was on the left and the map of the cited journals was on the right. Collectively, there were three main citation paths on the current map. The published studies mainly targeted the journals in three fields: i) molecular biology and immunology; ii) medical and clinical areas; and iii) neurology, sports, and ophthalmology whereas the most cited publications originated from the journals of molecular biology and genetics.

**Table 4:** Top 10 journals with most publications in the field of targeted immunotherapy for MG.

Rank	Journal	Number of publications	Number of citations	Citations of per article	H-Index
1	Journal of Neuroimmunology	44	636	15.05	15
2	Muscle Nerve	41	1246	31.61	19
3	Annals of the New York Academy of sciences	27	556	20.85	14
4	Frontiers in Immunology	26	310	12.38	11
5	Frontiers in Neurology	19	99	5.42	6
6	Neurology	19	981	52.37	13
7	Current Opinion in Neurology	15	688	46.2	13
8	Clinical and Experimental Immunology	14	471	33.71	11
9	Journal of Immunology	13	600	46.31	11
10	Journal of Neurology	13	240	18.85	7

### Analysis of authors and co-cited authors

The number of research papers published by an author may translate the contribution to the research in the field. A total of 200 researchers authored 824 articles. The top 10 most productive authors in the field are presented in **Table 5**. Tzartos SJ [47 (5.70%)] had the highest number of publications, followed by Vincent A [21 (2.55%)], Howard JF [21 (2.55%)], Evoli A [19 (2.31%)], and Nowak RJ [19 (2.31%)]. Additionally, the CiteSpace software analyzed the author’s co-citation. Nevertheless, the centrality of the top 10 authors was not high and was <0.1 for each author, and a small number of links were observed on the network map, which indicated that there was little collaboration between different researchers in this research field.

**Table 5:** The top 10 most productive authors contributed to targeted immunotherapy for MG.

Rank	Author	Number of publications	Number of citations	Citations of per article	H-Index
1	Tzartos SJ	47	896	20.61	12.5
2	Vincent A	21	1100	49.13	16
3	Howard JF	21	832	41.85	13
4	Evoli A	19	880	47.74	14
5	Nowak RJ	19	651	36	12
6	Kaminski HJ	18	435	26.28	11
7	Mantegazza R	17	617	38	12
8	Berrih aknin S	15	356	24.67	11
9	De Baets MH	15	270	19	8
10	Martinez Martinez, P	14	245	19.07	9

### Analysis of references with citation burst

**Figure 8:**
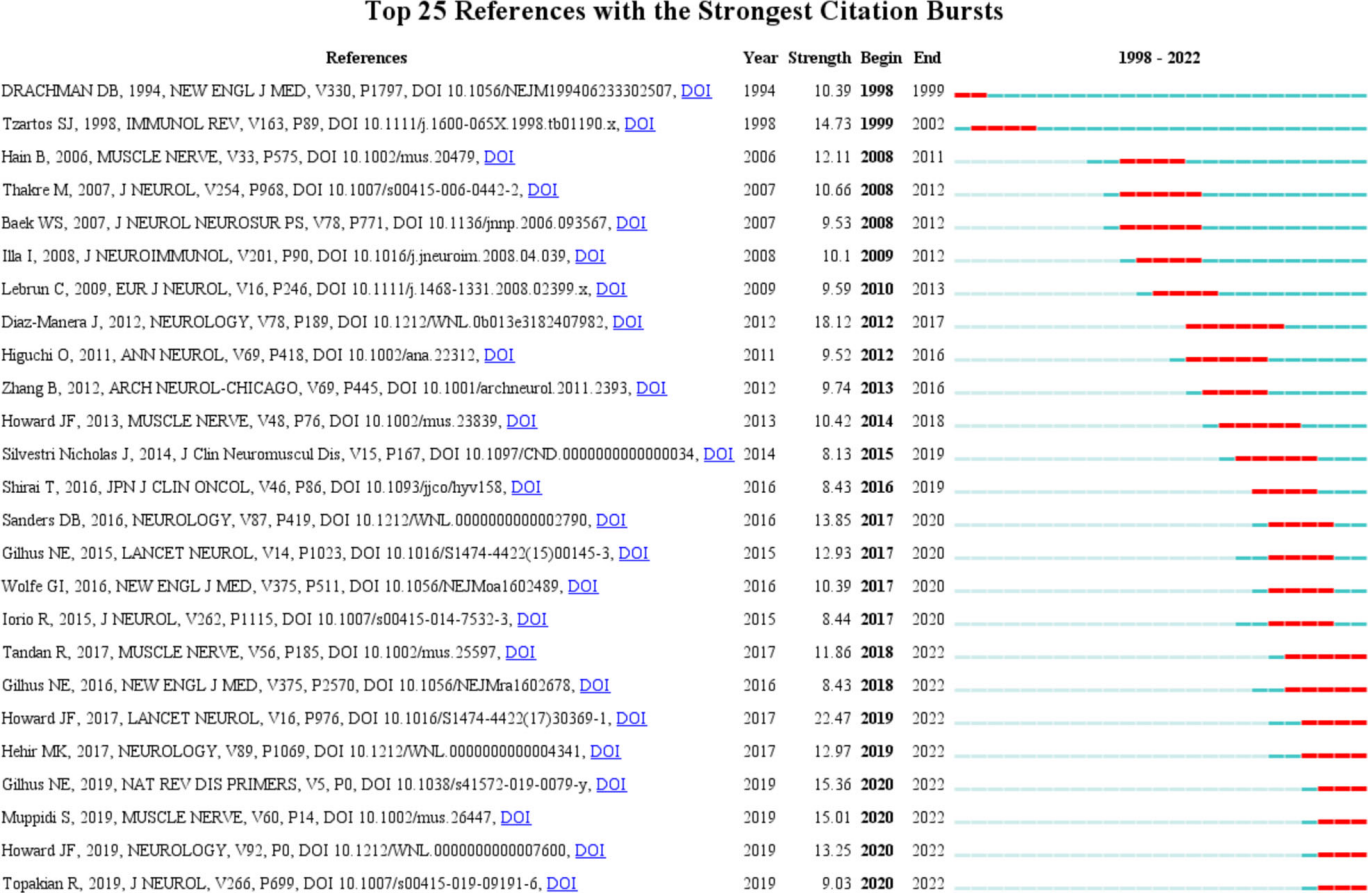
The top 25 references with the strongest citation bursts. The red segment represents the begin and end year of the burst duration.

The top 10 original articles relevant to targeted immunotherapy for MG with the most citations are summarized in **Table 6**. These selected articles span from 2000 to 2017. The most highly cited paper was published in 2006 and was written by Pescovitz, MD with 392 citations (37). The second co-cited paper was written by Vincent, A with 359 citations (38). The third co-cited paper was published by Zimmer, L with 354 citations (39). Burst detection, an algorithm developed by Kleinberg, was considered a tool to identify research frontiers or emerging trends in research over time (36). In our study, the burst detection algorithm was used to determine key references and keywords for targeted immunotherapy for MG. The blue line represented the period, and the red line indicated the duration of the reference burst occurrence. Among these references, REGAIN study had the strongest burst reference during the period from 1998 to 2022. Howard JF published the article, and its strength value was 22.47 (40). This study further assessed the efficacy and safety of eculizumab, a terminal complement inhibitor, in anti-acetylcholine receptor antibody-positive refractory patients using a phase 3 trial. This finding provided a novel perspective for the further development of targeted immunotherapy for MG. Citation bursts determined the frequency of citations for a reference over a period and the establishment of findings in this field. The CiteSpace was used (Selection Criteria: Top 25; The Number of States: 2; Minimum Duration: 2) to obtain 165 references with the most robust citation bursts for the targeted immunotherapy for MG. **Figure 8** shows the top 25 among them. The first burst of co-cited reference began in retrieval time (1998), which was a review on MG. Currently, 8 of the 25 references were still in the burst. Therefore, targeted immunotherapy for MG-related research fields may advance in the future. We also performed the reference co-citation analysis (**Supplementary Figure S1**) and the cluster view map (**Supplementary Figure S2**) by CiteSpace, **Supplementary Figure S1** displays the first author, and the year of the co-citations of references. Each circle represents a reference. The link between the two circles represents two references cited in the same article among the 824 articles (citing articles) retrieved in this study. A cluster view map is conducted if the two articles have many similar references and are often homogeneous. The largest eight clusters extracted from the references of the 824 citing articles are shown in **Supplementary Figure S2**, including #1 myasthenia gravis treatment, #2 muscle, #3 musk antibodies, #4 orphan drugs, #5 versus-host disease, #6 pembrolizumab, #7 complementary peptide, #8 complement activation. The total Modularity Q (0.7908) and Mean Silhouette (0.907) values were both greater than 0.5, suggesting that the cluster quality was reasonable.

**Table 6:** The top 10 co-cited references of targeted immunotherapy for MG.

Rank	Title	Journal	Country	Author	Years	Number of citations
1	Rituximab, an anti-CD20 monoclonal antibody: History and mechanism of action	American Journal of Transplantation	USA	Pescovitz, MD	2006	392
2	Myasthenia gravis	Lancet	England	Vincent, A	2001	359
3	Neurological, respiratory, musculoskeletal, cardiac and ocular side-effects of anti-PD-1 therapy	European Journal of Cancer	Germany	Zimmer, L	2016	354
4	Imbalance of regulatory T cells in human autoimmune diseases	Immunology	Austria	Dejaco, C	2006	254
5	Safety and efficacy of eculizumab in anti-acetylcholine receptor antibody-positive refractory generalised myasthenia gravis (REGAIN): a phase 3, randomised, double-blind, placebo-controlled, multicentre study	Lancet Neurology	USA	Howard, JF	2017	226
6	Acetylcholine receptors and myasthenia	Muscle & Nerve	USA	Lindstrom, JM	2000	202
7	Long-lasting treatment effect of rituximab in MuSK myasthenia	Neurology	Spain	Diaz-Manera, J	2012	194
8	Myasthenia gravis: An emerging toxicity of immune checkpoint inhibitors	European Journal of Cancer	Australia	Makarious, D	2017	146
9	A randomized, double-blind, placebo-controlled phase II study of eculizumab in patients with refractory generalized myasthenia gravis	Muscle & Nerve	USA	Howard, JF	2013	131
10	Rituximab treatment of myasthenia gravis: a systematic review	Muscle & Nerve	USA	Tandan, R	2017	121

### Analysis of keywords

**Figure 9:**
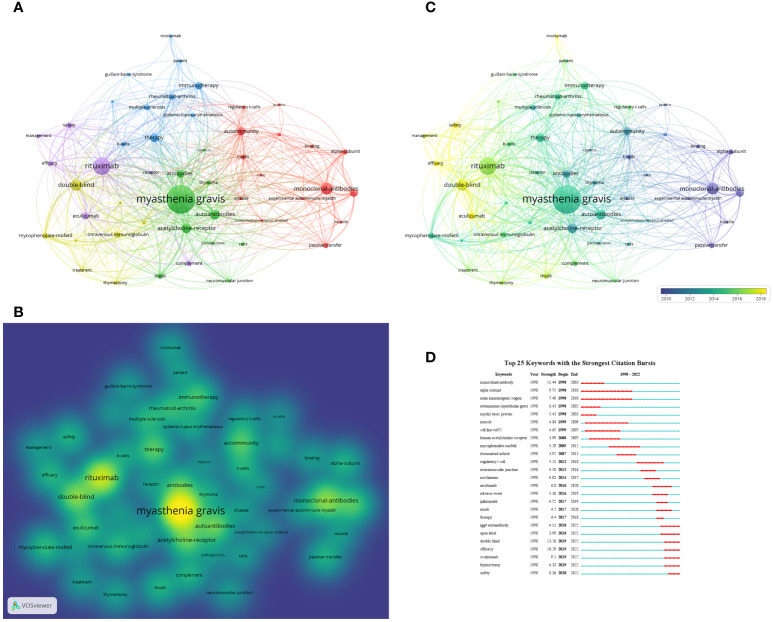
**(A)** Network visualization of keywords based on VOSviewer. In this network map, keywords with close relationship are assigned to one cluster with the same color. All the keywords could be divided into five clusters: cluster 1 (red nodes), cluster 2 (green nodes), cluster 3 (blue nodes), cluster 4 (yellow nodes) and cluster 5 (purple nodes). **(B)** Density visualization of keywords based on VOSviewer. **(C)** Overlay visualization of keywords based on VOSviewer. The nodes marked with purple or blue color represent the keywords that appeared relatively earlier, whereas keywords coded with yellow color represents the current research focuses. **(D)** The top 25 keywords with the strong citation bursts in articles related to targeted immunotherapy for MG.

In addition to references, keywords can offer readers information about the research topics and methodologies of the publications, and analysis of keywords co-occurrence is often employed to detect the research hotspots and directions in the research field. The network visualization map was generated for keywords with the value of co-occurrence greater than 20 times. As shown in **Figure 9A**, there were 50 nodes, 839 links, and a total link strength of 4293 on the visualization map, the “myasthenia gravis” at the center of node, followed by “rituximab”. The density visualization map of the keywords is illustrated in **Figure 9B**, the top three keywords with the greatest number of occurrences are “myasthenia gravis” which appears 309 times; followed by “rituximab” and “monoclonal-antibodies” which appears 171 times and 111 times, respectively. The overlay visualization map is shown in **Figure 9C**, summarizing the keyword occurrences from a time zone perspective.

### Burst keywords

The CiteSpace was used to detect burst keywords to determine the hotspots and research frontiers over time. The burst keywords are terms cited frequently over a period. The top 25 keywords with the strongest citation bursts are presented in **Figure 9D**. The blue line represents the time from 1998 to 2022, while the periods of each burst keyword are plotted by the red line. The keywords that had citation bursts after 2018 were “igg4 autoantibody’’ (2018-2022, strength of 4.11), ‘‘open-label’’ (2018-2022, strength of 3.99), ‘‘double-blind’’ (2019-2022, strength of 13.26), ‘‘efficacy’’ (2019-2022, strength of 10.29), “eculizumab” (2019-2022, strength of 9.1), “thymectomy” (2019-2022, strength of 4.33), and “safety” (2019-2022, strength of 8.36). In particular, the burst of these six keywords including “open-label”, “double-blind”, “efficacy”, “eculizumab”, “thymectomy” and “safety” is still in progress.

## Discussion

The current study was the first to use bibliometric methods to measure research trends on targeted immunotherapy for MG from 1998 to 2022. Unlike systematic reviews and scoping reviews, bibliometric analysis has become a powerful tool for summarizing the current status of knowledge and predicting future trends (41, 42). Based on information science, computer science, scientometrics, and applied mathematics, the visualization map exhibited specific knowledge domain and structural relationships, which were generated by the VOSviewer or CiteSpace (32, 34, 43). After excluding 169 studies that did not meet the inclusion criteria, 824 publications in 323 journals with 18346 co-cited references from 1084 institutions in 59 countries/regions were eligible for the analysis. Subsequently, bibliometric and visualization analysis tools were used to identify the main publications and citations, contributing countries, institutions, authors, funding agencies, knowledge base, research hotspots, and emerging topics.

From 1998 to 2022, the total number of publications on MG was 7672, and the total number of publications on targeted immunotherapy for MG was 824, which accounting for 10.74%. **Supplementary Figure S3** shows that the number of publications on MG has maintained a steady growth before 2020. After 2020, the annual number of publications on MG exceeded 500 for the first time. **Supplementary Figure S4** shows the annual publication output on targeted immunotherapy for MG dramatically increased after 2017. The reason for this phenomenon is the REGAIN study was published (40) and the US Food and Drug Administration (FDA) approved eculizumab for use in the refractory AChR-GMG in 2017. Although the growth time point are different, it can be seen that the number of publications has shown a continuous growth trend over time, whether the field of MG or targeted immunotherapy for MG. However, the pathogenesis, diagnostic methods, and biomarkers of MG are also the research hotspots in the MG field. Therefore, the publication output trend of MG targeted immunotherapy can partially represent the trend of MG.

In recent years, targeted immunotherapy has gradually entered the view of neuroimmunology specialists. The research on targeted immunotherapy for autoimmune diseases has also increased over time, including NMOSD (neuromyelitis optica spectrum disorders), MS (multiple sclerosis), MG, etc. We searched the publications on targeted immunotherapy for NMOSD and MS. **Supplementary Figure S5** shows that the NMOSD targeted immunotherapy have been first published since 2004, the cause of this phenomenon is the discovery of AQP4 (aquaporin 4) antibody in 2005 (44), NMOSD was independent from MS, and the public had a new understanding of the pathogenesis of NMOSD. Since then, biological agents for various targets on its pathogenesis have been developed. **Supplementary Figure S6** shows that the publication outputs of targeted immunotherapy for MS is highly than NMOSD and MG and shows a continuous growth trend. This is due to the high incidence rate, heavy disease burden, high disability rate of MS, the R & D cost of DMTs (disease-modifying therapies) is high corresponding. For example, the approval and listing of ocrelizumab, natalizumab and ofatumumab have played a role in promoting the DMTs of MS.

Comparing the number of publications on targeted immunotherapy for NMOSD, MS and MG, they all show an increasing trend, but each disease has its own unique increasing trend. This is because the pathogenesis, the time of significant breakthrough was achieved and investment in different diseases are different. For example, MS is mediated by cellular immunity, while NMOSD and MG are caused by humoral immunity and the production of AQP4 antibody and AChR antibody respectively. In conclusion, the MG has a special growth trend compared with other diseases.

A general upward trend was found in the number of targeted immunotherapies for MG-related publications, indicating that this field was actively researched in recent years. Two phases, including a slow growth period (1998-2016) and a rapid growth period (2017-2022) were noticed. The number of publications exhibited a stable increasing trend from 1998 to 2016, but the increase was not apparent because the average annual publication volume did not exceed 35 articles. After 2017, the annual publication output dramatically increased which peaked in 2020, and this stage accounted for 47% of the total publications. The growth trend of citations was consistent with the publications. This phenomenon may have been connected to the significant events in this field, Firstly, REGAIN study entitled “Safety and efficacy of eculizumab in anti-acetylcholine receptor antibody-positive refractory myasthenia gravis: a phase 3, randomized, double-blind, placebo-controlled, multicenter study” (40) was published in 2017. The safety and efficacy of targeted biological agents in generalized myasthenia gravis (GMG) were confirmed for the first time. Second, based on the above findings, the US Food and Drug Administration (FDA) approved eculizumab for use in the refractory AChR-GMG in 2017. Before that approval, targeted biological agents were widely used in the treatment of other autoimmune diseases (13, 45–47). After the FDA approval of eculizumab to treat refractory AChR-GMG in 2017, targeted immunotherapy for MG has attracted the enthusiasm and attention from pharmaceutical companies and researchers. Phase II and phase III clinical trials on targeted immunotherapy for MG have been supported by complete funding, and several new targets are also being exploited (48, 49). Therefore, the number of publications and citations on targeted immunotherapy for MG has been increasing after 2017. Although only 29 articles in the first quarter of 2022 were retrieved, the number of articles in 2022 will reach a new peak according to the current growth trend. Improved diagnosis of the disease can seemingly contribute to increased disease incidence and population. From 1998 to 2022, the detection technique of autoantibodies is diverse persistently, and the sensitivity and specificity have been improved continuously. With the improvement of diagnostic methods, the diagnosis rate of MG has been improved, which increased the prevalence greatly. The development and application of targeted immunotherapy not only reduces the side effects of hormones and traditional immunosuppressants, but also further reduces the MG relapse and MG crisis, which greatly promotes the demand of MG patients for targeted immunosuppressants. The development of these new targets and successful clinical trials in MG will provide more treatment options for MG patients.

The distribution of contributing countries/regions and institutions showed some characteristics. As shown in **Table1** and **Figure 3**, the United States ranked first in the number of publications, citations, and h-index in this field. Furthermore, the United States had a solid foundation in the biomedical field for a long time. The United States received a large amount of financial support and showed a sufficient reserve of researchers and institutions from funding and institution analyses. The top three funding agencies were the National Institutes of Health (NIH), the United States Department of Health and Human Services (HHS), and the NIH National Institute of Neurological Disorders and Stroke (NINDS) and were from the United States. Four of the top 10 institutions with the most publications were also from the United States, indicating that the United States is the most influential country in this research field, which is far ahead of other countries. Besides the USA, China exhibited an increasing trend in advancement in this area. Much attention has been paid to this research area in the past years in China. Following the development of the Chinese economy, the healthcare needs of the general population are on the rise, and the financial support for the medical and health fields is also gradually increasing, especially focusing on molecular biological treatment options. Nevertheless, the citations and h-index were low in China when compared with other countries. Although the economic development is rapid in China, the advancement in the biomedical field was relatively behind and the groundwork was weak. Due to the large population in China, the medical insurance is challenged because one-year treatment with eculizumab costs approximately $500,000. Further, high research and development (R&D) costs and clinical expenses limited the clinical promotion and application of targeted immunotherapy to some extent. Although the number of publications from China in international journals has significantly increased, high-quality research papers have been published infrequently in top-grade journals (50, 51). However, the matter has attracted great attention from policymakers, and they encouraged researchers to improve the research quality, not the quantity of research (52). Additionally, the United States had the largest international cooperation in this field, followed by China (**Figure 4**). But collaboration between China and other countries was not strong. The developing countries should encourage their institutions to participate in research, strengthen collaboration, promote the advancement of related fields, and publish high-quality articles.

An analysis of journals and co-cited journals can provide a wealth of information for researchers to choose the best journal to submit their manuscripts (29, 53). There were 28% of articles published in the top 10 journals (**Figure 6** and **Table 4**). The most productive journals in this field were the *Journal of Neuroimmunology* (IF=3.478), followed by *Muscle Nerve* (IF=3.217) and *Annals of the New York Academy of Sciences* (IF=5.691). Due to the rare nature of MG, the option for journals is narrow. Although the impact factor of the top 3 journals was not high, *Muscle Nerve* is a professional journal in the MG field and all other journals are comprehensive in immunology or neurology. Researchers can focus on these journals to know about research trends and frontiers in targeted immunotherapy for MG. In addition, when submitting manuscripts, researchers can find the most suitable journals for timely processing, avoiding delays in time of the study. **Figure 7** shows that publications in “molecular biology, genetics” are often cited in “medicine, medical and clinical”, indicating that current research focuses more on clinical research and translational research.

In our analysis, Vincent A scored the highest citations and h-index. Further, Vincent A and his research team had the highest research strength and influence. They published important findings in this field when compared to others.

### Knowledge base

The more frequently an article is cited, the more important it is perceived in a specific field. Therefore, the most cited publications or influential literature can be regarded as a knowledge base in a particular field (54). As shown in **Table 6**, among the top 10 cited articles, there were 6 reviews, 2 randomized controlled trials (RCT), and 2 clinical research. From the time of publication perspective, 4 articles were published between 2000 to 2010 (early phase), which were reviews, 2 articles were published between 2011 to 2015 (middle phase) that were clinical research, and 4 articles were published between 2016 to present (recent phase), which had 2 reviews and 2 clinical studies.

The 4 reviews published in the early phase mainly described the pathogenesis, diagnosis, and treatment of MG, and these reviews played a landmark role in elucidating the mechanism of pathogenesis and diagnosis of MG. The article titled “Rituximab, an anti-CD20 monoclonal antibody: History and mechanism of action” was published by Pescovitz MD (37), which was the most cited paper from the analysis. This article mainly reviewed the history, pharmacokinetics, and potential mechanism of action of rituximab (RTX). After Zaja F first reported that RTX could be used for the treatment of GMG patients in 2000 (23), targeted immunotherapy was first envisioned by researchers. Since then, case reports and small-sample studies on RTX treatment in refractory MG have been endlessly streaming (55–58). Although it was not supported by advanced evidence-based medical studies, it has been widely applied in the clinical field, and these studies reflected the effectiveness of RTX in patients with anti-Musk positive and some anti-AChR positive MG (59, 60). The second co-cited paper titled “myasthenia gravis” was published by Vincent A (38), and was published in *Lancet* in 2001. This article summarized the epidemiology, clinical characteristics, classification, pathophysiological parameters, treatment, diagnosis, and differential diagnosis of MG. As a high-quality review in the field of MG, it served as a very important reference value for the targeted immunotherapies for MG. The third co-cited paper “Imbalance of regulatory T cells in human autoimmune diseases” was published by Christian Dejaco (61). This article described the unique role of Treg cells in autoimmune diseases, which exhibited their inhibition function *in vitro* in a contact-dependent manner and preferentially expressed high levels of CD25, forkhead, and winged-helix family transcription factor forkhead box P3 (FOXP3) (Tregs). In autoimmune diseases, altered Tregs and insufficient suppression of inflammation were thought to be critical factors for disease development and persistence.

During the middle phase, “A randomized, double-blind, placebo-controlled phase II study of eculizumab in patients with refractory generalized myasthenia gravis” was published by Howard JF (62). This study outcome suggested that the overall change in mean total QMG score was significantly different between eculizumab and placebo therapies (P<0.0001). Another clinical study on RTX in Musk MG patients was published in *Neurology* in 2012 (63). Previous articles were all case reports or small sample studies on RTX in the treatment of MG (64, 65). But in this article, anti-AChR positive MG patients were used as controls. The study participants were prospectively followed for up to 31 months and compared with anti-AChR-positive MG patients. All the anti-Musk-positive groups achieved remission or showed minimal manifestations status (MMS), prednisone doses were significantly reduced, and concomitant immunosuppressants were withdrawn. At the last follow-up, Musk antibodies were negative in 3 of these patients and showed a decrease of over 80% in the other three patients. This study described better treatment options for patients with anti-Musk-positive MG. These two studies developed a new perspective on targeted biological agents for the treatment of MG patients. It was translational findings between the early stage and recent stage and provided evidence for more targeted biological agents in treating MG in the future.

Four articles published during the recent stage were in the direction of targeted immunotherapy for MG, which showed that the theory and mechanism of targeted immunotherapy have been mature, which have gradually entered the clinical transformation and application stage. Especially, the REGAIN study published by Howard, JF in *Lancet Neurology* in 2017, which was the phase III clinical study on eculizumab in refractory GMG. Since the publication of this study, articles on targeted immunotherapy for MG exhibited an explosive growth trend and more and more targeted biological agents were investigated. For example, efgartigimod (a FcRn antagonist) completed phase III clinical trials and was approved by the FDA for the treatment of anti-AChR positive GMG (11). The citations of “rituximab treatment of myasthenia gravis: a systematic review” published in 2017 were ranked in the top 10 (55). Although various targeted biological agents for the treatment of MG are under development, the popularity of RTX in the treatment of MG is still advancing. Several reasons may have accounted for this phenomenon. First, the results of RTX in different studies were not consistent. Second, the RTX has been administered clinically for almost 2 decades, while other targeted biological agents only have been used to treat MG in recent years (66, 67). Compared with other targeted biological agents, the RTX therapy has been adopted for a long and had long-term safety data. Third, the affordability, accessibility, and availability of the RTX are generally good, and the cost is bearable by the patients. In 2020, Brauner et al. published an article in *JAMA neurology*, which compared the therapeutic effect of the RTX in new-onset and refractory generalized MG patients (68). Surprisingly, the RTX was more favorable in new-onset generalized MG (69, 70), and the RTX performed better than the conventional immunosuppressant therapy. These findings showed a relatively greater benefit of RTX earlier in the disease course. Therefore, a placebo-controlled randomized trial to corroborate these findings is warranted.

Although most references burst has ended, several reference bursts are still ongoing, and most of these references focused on the clinical studies of targeted immunotherapy for MG, such as RTX, eculizumab, and efgartigimod, indicating continuous advancement in recent years (**Figure 8**).

### Research hotspot

Keywords can reflect the research hotspots and frontiers in a specific research field. In addition to “myasthenia gravis”, the most representative keyword was “rituximab” (**Figure 9**). The keywords with a strong link with RTX were “safety”, “efficiency”, “therapy”, and “double-blind”, which was consistent with the results of references co-cited. RTX is the first targeted biological agents which was used in the clinical practice for MG. For a long time, it is also the only targeted biological agents for MG patients. Therefore, the research on RTX in the targeted immunotherapy field is most. From the keywords with the strong link with RTX, researchers are still concerned about the effectiveness and safety of RTX. Because of long-term off-label use, there urgently need for conduct high quality double-blind RCT. The publication of the REGAIN study in 2017 laid the foundation for the safety and efficacy of targeted biological agents in MG for the first time, which has evoked enthusiasm from researchers. The next focused keyword was “double-blind”, which showed that a growing number of clinical studies with high-quality evidence-based medicine have been conducted recently. The safety and efficacy of many targeted biological agents have been gradually confirmed. Therefore, researchers are encouraged to develop more new target molecules in the future. Interestingly, the keywords were “mice”, “experimental autoimmune MG”, “regulatory T -cells”, “T-cells”, and “alpha subunit” between 2010 and 2012 (**Figure 9C**). These keywords mainly focused on the basic research of MG. The keywords were “patient”, “therapy”, “intravenous immunoglobulin”, “mycophenolate-mofetil”, “thymus”, “thymoma”, and “thymectomy” between 2012 and 2016,. The keywords in 2016-2018 were mainly “eculizumab”, “rituximab”, “nivolumab”, “double-blind”, “safety”, “efficiency”, and “management”, which focused on the targeted immunotherapy for MG with high-quality clinical research. The basic research on targeted immunotherapy has been relatively mature, and multiple immune targets have been identified. With the basic research to clinical translation, the investment in new drug research has increased, and researchers has gradually focused to clinical research.

In addition, the CiteSpace was used to analyze keywords, which were used to identify the research hotspots and frontiers of research during the period. The evolution of burst keywords over the past decade demonstrated the continued progress in the field of targeted immunotherapy for MG. The result was consistent with the VOSviewer (**Figure 9D**). The “double-blind” was also the strongest burst keyword. Several keywords are still in the burst presently, suggesting the safety and efficacy of targeted immunotherapy and the development of high-quality RCT is also the research hotpots. There is a need for increased research efforts in this area so that MG patients of different types will have more treatment options, which is conducive to individualized precision therapy.

### Strengths and limitations

To the best of our knowledge, this was the first study using the bibliometric method to summarize the status and development of the targeted immunotherapy for MG. To comprehensively evaluate the existing literature, the data were analyzed using two bibliometric tools (CiteSpace and VOSviewer) and an online platform. Despite the above‐mentioned strengths, several limitations are unavoidable, First, there are diverse factors that can affect the number of publications, both known and unknown. It is very difficult to obtain the overall funds data in different countries from the WoSCC database (the specific amount of fund support is not available from the WoSCC database). Moreover, many countries lack national epidemiological data in MG field, which may cause statistics bias. Therefore, our study adopted bibliometric analysis methodology, we only compared the number of publications in different countries, and through this important indicator to reflect the research status of different countries. This method has many limitations obviously, but it is also a feasible method at present. Second, the database selection bias was that all literatures included in this study were downloaded from the WoSCC. The relevant studies deposited in other databases might have been missed. Finally, our literature search was only dependent on the English language, hence our analysis may have excluded articles reported in non-English.

## Conclusion

Taken together, the current study summarized the global research trends concerning the targeted immunotherapy for MG. The outcome of the study demonstrated that the USA is leading ahead in both the sum of publications, total citation frequency, and funding in this field. Along with improving life quality of MG patients, high efficiency, rapid onset, less side effects and good compliance have become new treatment needs. The MG treatment has entered a personalized precision treatment phase. Further exploration into new target molecules and conducting high-quality randomized controlled trials on existing biological agents are urgently needed to guide the future directions of immunotherapy research. Consequently, it is not difficult to predict that this field is likely to advance rapidly and more studies will be published in the future. Meanwhile, there are also multiple challenges for MG targeted immunotherapy in the future, such as the long-term effectiveness, safety, accessibility, and cost of biological agents. We should focus on providing precise treatment schemes for patients with different subtypes, which will help patients achieved the treatment goal rapidly, reduce the treatment burden and provide convenience for patients to the greatest extent.

## Data availability statement

The raw data supporting the conclusions of this article will be made available by the authors, without undue reservation.

## Author contributions

TC conceptualized the study, secured funding, and designed uniform procedures for data collection across the study centers. TC, ZL, YS, ZR, RW and SH contributed to the study design. YS, ZR, RW and SH contributed to collect and analyzing data. TC, YS, ZR, RW and SH contributed to the drafting of the manuscript. YT, XH and TG contributed to collecting data. All authors contributed to the critical revision of the manuscript for important intellectual content and provided approval of the final manuscript for submission.

## Funding

The authors disclosed receipt of the following financial support for the research, authorship, and publication of this article: This work was supported by the discipline innovation and development plan of Tangdu Hospital-major program of clinical research (Grant No. 2021LCYJ002), Key Research and Development Projects of Shaanxi Province (Grant No. 2021ZDLSF02-01).

## Acknowledgments

The authors greatly acknowledge Yafei Qin for the revision of the article and Liang Wang provided methodological support.

## Conflict of interest

The authors declare that the research was conducted in the absence of any commercial or financial relationships that could be construed as a potential conflict of interest.

## Publisher’s note

All claims expressed in this article are solely those of the authors and do not necessarily represent those of their affiliated organizations, or those of the publisher, the editors and the reviewers. Any product that may be evaluated in this article, or claim that may be made by its manufacturer, is not guaranteed or endorsed by the publisher.
