# Supplementary material for: Knowledge mapping of targeted immunotherapy for myasthenia gravis from 1998 to 2022: A bibliometric analysis
Source: Front Immunol. 2022 Sep 29;13:998217. doi: 10.3389/fimmu.2022.998217 (PMC9557176; doi:10.3389/fimmu.2022.998217)
Supplement: Supplementary file 1 [file DataSheet_1.docx]

Supplementary Material

## Supplementary Figure 1


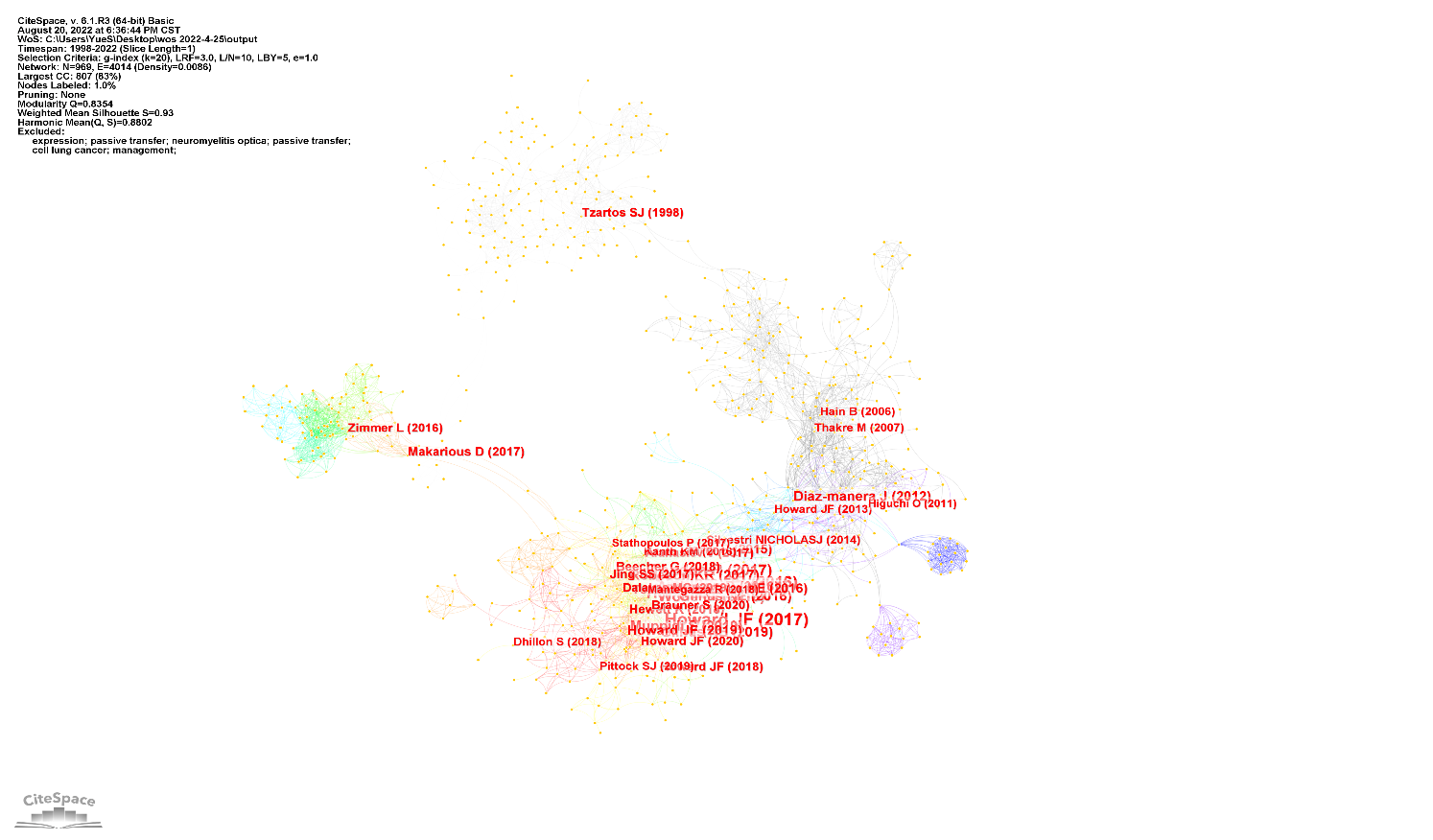


## Supplementary Figure 1: Visual analysis of reference co-citation. The nodes in the figure represent the co-citation literature, and the links between nodes represent the co-citation relationship. Large nodes are either highly referenced or erupted.

## Supplementary Figure 2


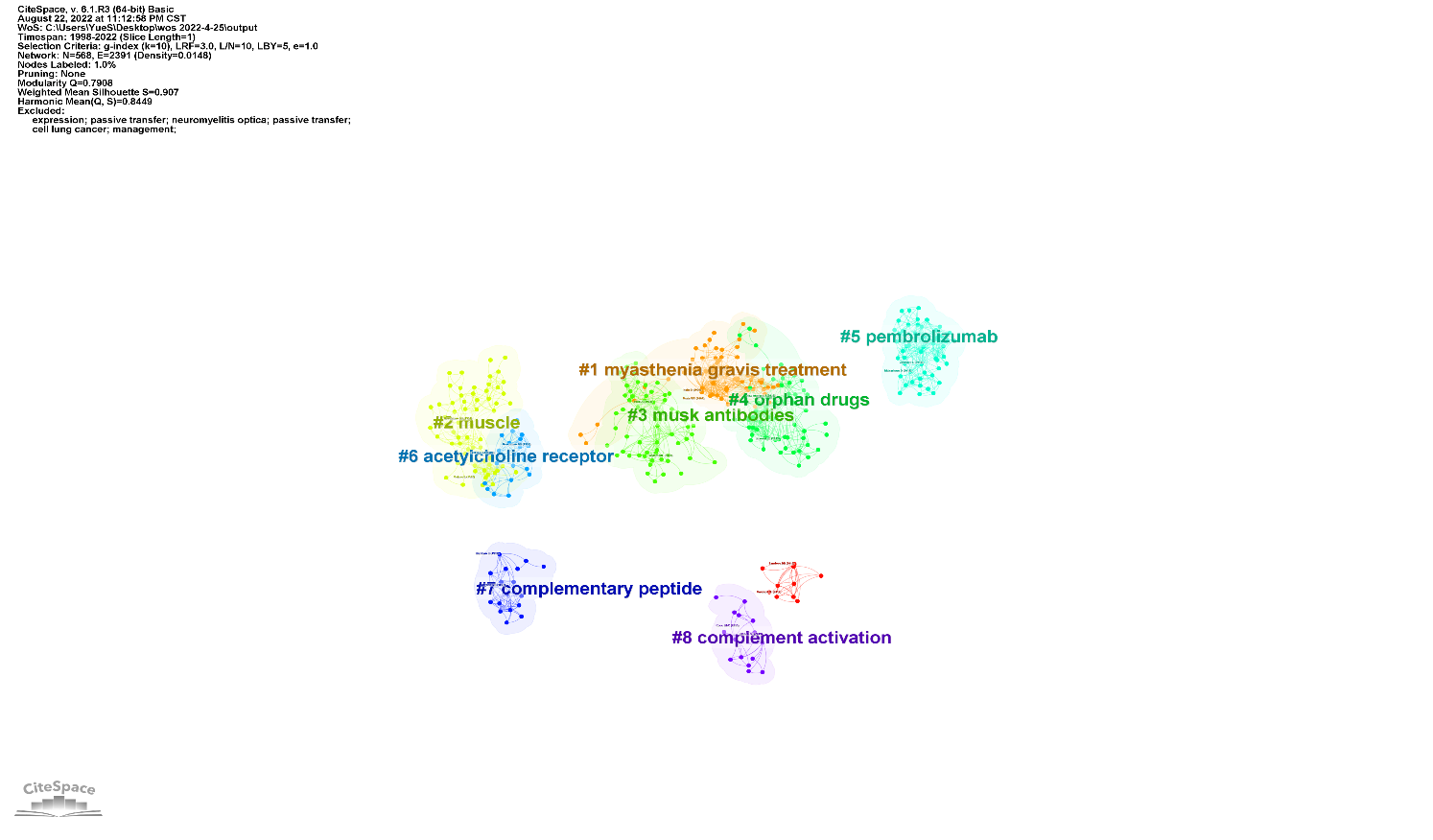


## Supplementary Figure 2: Visualization of cluster view map. All cluster labels were extracted from keywords of citing articles using the log-likelihood ratio algorithm.

## Supplementary Figure 3

##
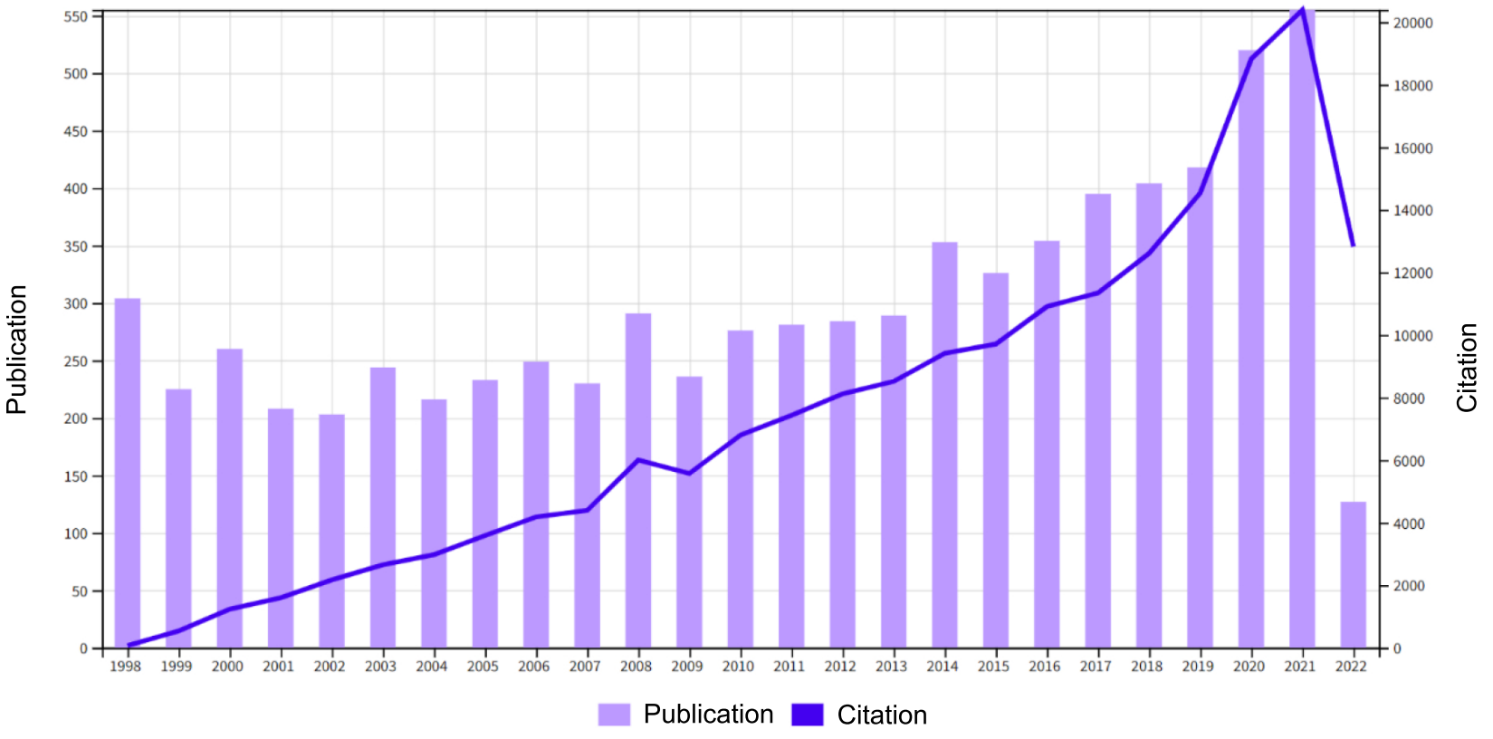


**Supplementary Figure 3: The overall growth trend of publications in myasthenia gravis field.**

## Supplementary Figure 4

**
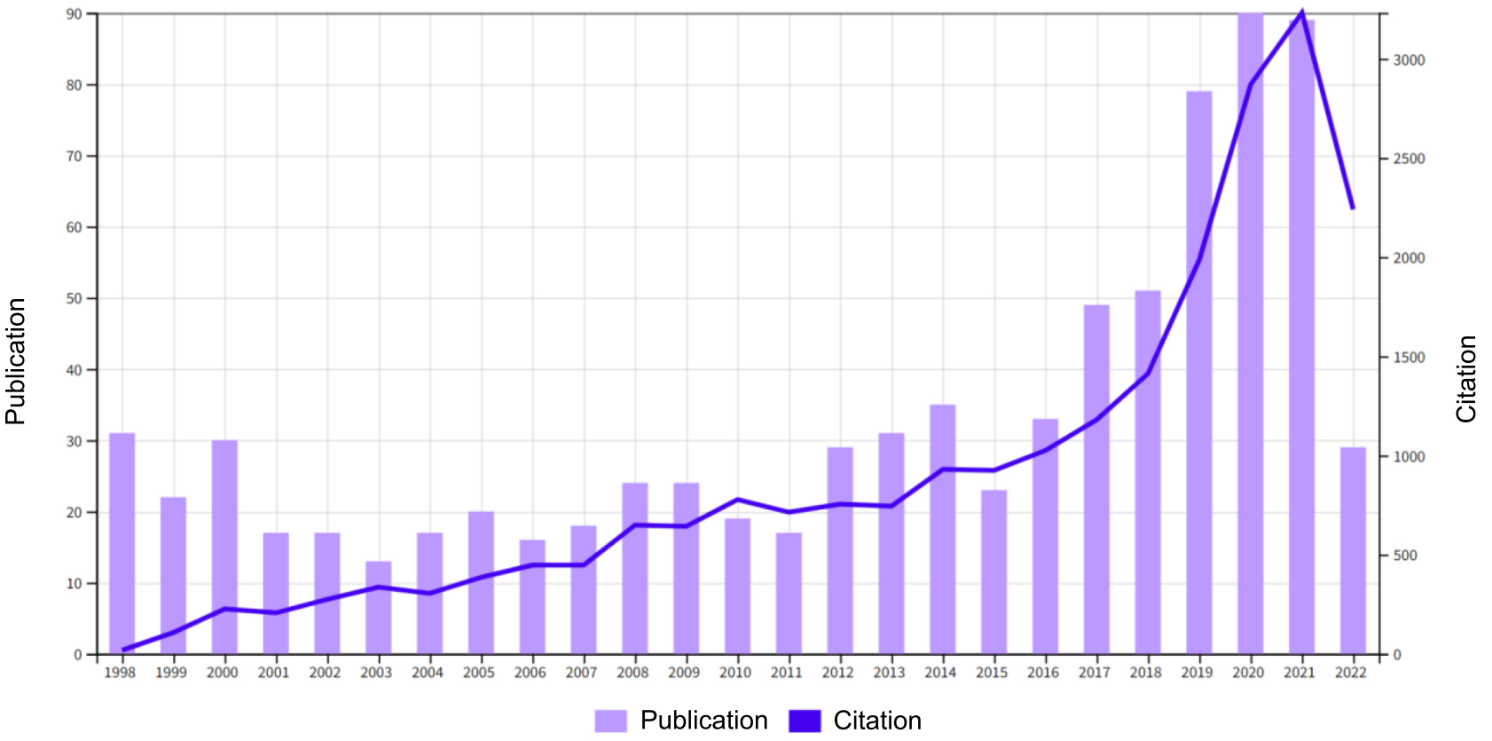
**

**Supplementary Figure 4: The overall growth trend of publications in targeted immunotherapy for myasthenia gravis.**

## Supplementary Figure 5


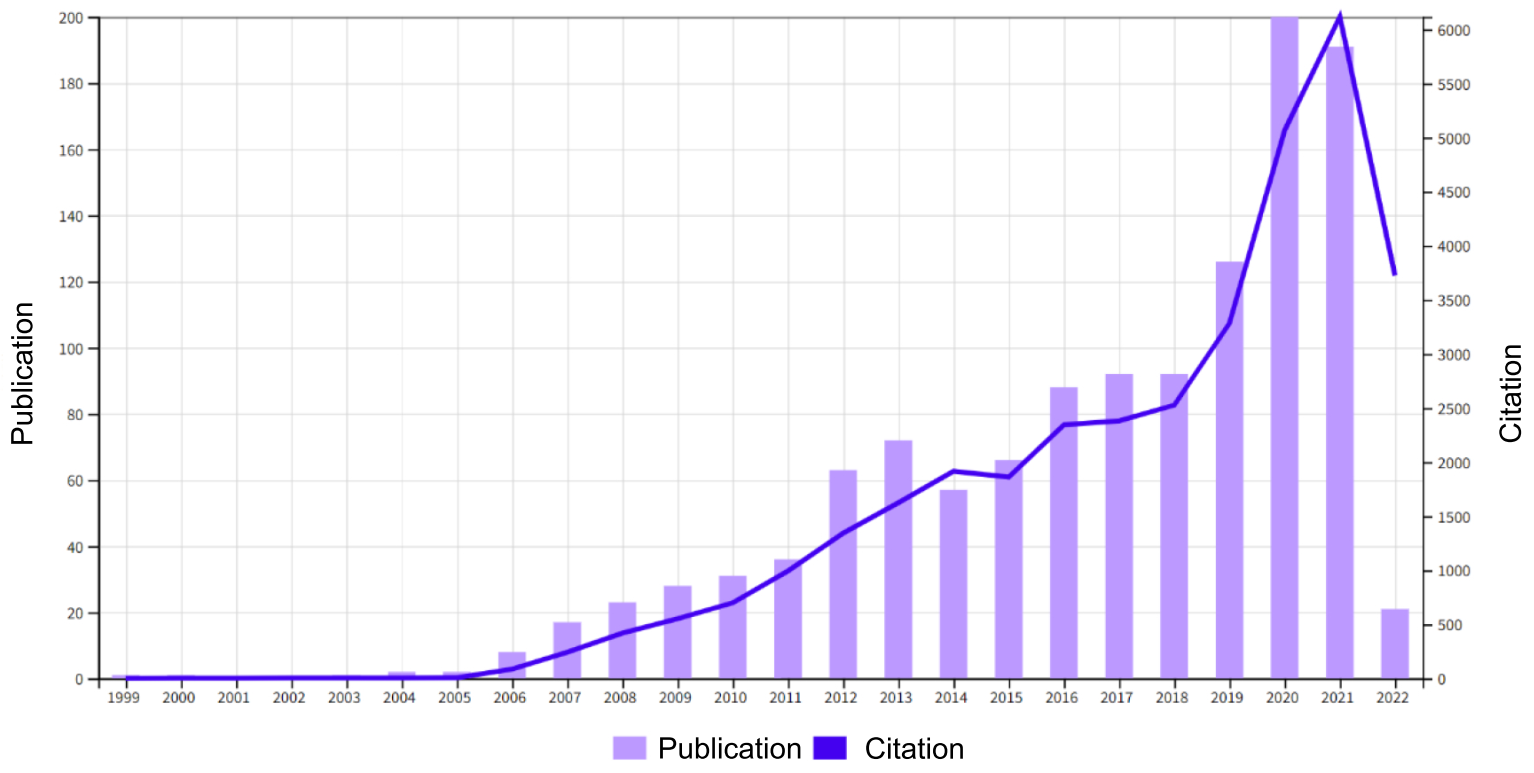


**Supplementary Figure 5: The overall growth trend of publications in targeted immunotherapy for neuromyelitis optica spectrum disorders.**

## Supplementary Figure 6


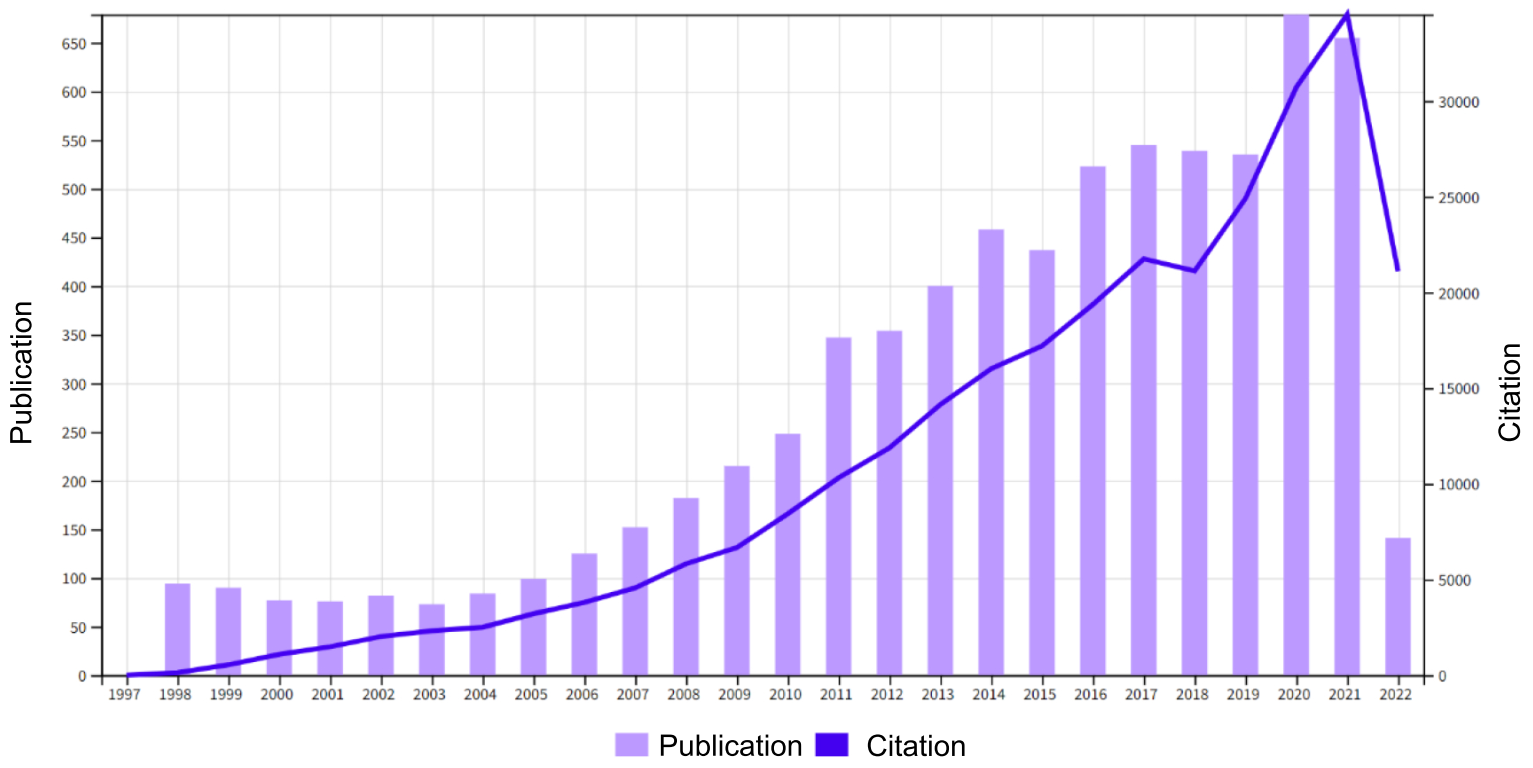


**Supplementary Figure 6: The overall growth trend of publications in targeted immunotherapy for multiple sclerosis.**
